# Recognition of Urdu sign language: a systematic review of the machine learning classification

**DOI:** 10.7717/peerj-cs.883

**Published:** 2022-02-18

**Authors:** Hira Zahid, Munaf Rashid, Samreen Hussain, Fahad Azim, Sidra Abid Syed, Afshan Saad

**Affiliations:** 1 Faculty of Engineering Science Technology and Management, Department of Biomedical Engineering and Department of Electrical Engineering, Ziauddin University, Karachi, Pakistan; 2 Faculty of Engineering Science Technology and Management, Department of Electrical Engineering and Department of Software Engineering, Ziauddin University, Karachi, Pakistan; 3 HESSA Project, US AID Program, Karachi, Pakistan; 4 Faculty of Engineering Science Technology and Management, Electrical Engineering Department, Ziauddin University, Karachi, Pakistan; 5 Faculty of Engineering Science Technology and Management, Department of Biomedical Engineering, Ziauddin University, Karachi, Pakistan; 6 Computer Science Department, Muhammad Ali Jinnah University, Karachi, Pakistan

**Keywords:** Sign language recognition, Pattern recognition, Machine learning, Deep learning, Urdu sign language, Pakistani sign language

## Abstract

**Background and Objective:**

Humans communicate with one another using language systems such as written words or body language (movements), hand motions, head gestures, facial expressions, lip motion, and many more. Comprehending sign language is just as crucial as learning a natural language. Sign language is the primary mode of communication for those who have a deaf or mute impairment or are disabled. Without a translator, people with auditory difficulties have difficulty speaking with other individuals. Studies in automatic recognition of sign language identification utilizing machine learning techniques have recently shown exceptional success and made significant progress. The primary objective of this research is to conduct a literature review on all the work completed on the recognition of Urdu Sign Language through machine learning classifiers to date.

**Materials and methods:**

All the studies have been extracted from databases, i.e., PubMed, IEEE, Science Direct, and Google Scholar, using a structured set of keywords. Each study has gone through proper screening criteria, *i.e.*, exclusion and inclusion criteria. PRISMA guidelines have been followed and implemented adequately throughout this literature review.

**Results:**

This literature review comprised 20 research articles that fulfilled the eligibility requirements. Only those articles were chosen for additional full-text screening that follows eligibility requirements for peer-reviewed and research articles and studies issued in credible journals and conference proceedings until July 2021. After other screenings, only studies based on Urdu Sign language were included. The results of this screening are divided into two parts; (1) a summary of all the datasets available on Urdu Sign Language. (2) a summary of all the machine learning techniques for recognizing Urdu Sign Language.

**Conclusion:**

Our research found that there is only one publicly-available USL sign-based dataset with pictures versus many character-, number-, or sentence-based publicly available datasets. It was also concluded that besides SVM and Neural Network, no unique classifier is used more than once. Additionally, no researcher opted for an unsupervised machine learning classifier for detection. To the best of our knowledge, this is the first literature review conducted on machine learning approaches applied to Urdu sign language.

## Introduction

Everything in our world is imperfect, and there is no place for idealism, and many scientific data and figures demonstrate this. In the same way, humans are neither flawless nor ideal. Some people are born differently than others. We describe them as impaired since they are distinct, but in truth, they are unique and have particular requirements. It is estimated that over 72 million people worldwide (World Federation of the Deaf, 2022) have hearing impairment difficulties, with approximately 10 million people in Pakistan (Pakistan Association of the Deaf, 2022b) being deaf, as per the International Federation of the Deaf. There is no all-encompassing international system that provides a comprehensive manner for deaf people to talk with one another worldwide. Since the beginning of time, visual communication has conveyed information. Generally, various new types of sign languages are being used worldwide. A sign language is a way of communication that, instead of using sonically transmissible sound patterns, uses visually transmis (Disabled World, 2017) smoothly. To communicate effectively between the deaf community and the general public without paper and pencil, there are a variety of sign languages available in different countries (U. S. Department of Justice, 2020), including American Sign Language, British Sign Language, Spanish Sign Language, and probably sign languages throughout every country. Even if you are not fluent in sign language, you have almost certainly come into contact with it, either through witnessing it in action or through using a translator at a seminar or a performance. There is still more sign language than strikes the eye, and several dialects other than American Sign Language (ASL) are used for sign language communication. It is estimated that around 60 sign languages are recognized and utilized worldwide (Elakkiya, 2020). According to the National Institute on Deafness and Other Communication Disorders (NIDCD), ASL is “a complete and complex language that includes signals generated by moving the hands in conjunction with facial expressions and body postures”. It is more than just a translation of English into hand gestures; it has grammar and pronunciation norms and can handle varied ethnicities and accents (American Sign Language, 2021).

Furthermore, there is a lot of reported in different languages like Chinese (Jiang et al., 2020), American (Zafrulla et al., 2011), or Indian (Gupta & Kumar, 2021) that demonstrate that there is much work has been done on sign language recognition systems globally. The diversity of sign languages seen throughout the world indicates that local and regional language and culture are significant elements in the evolution of sign language, as is true of the development of any spoken language, regardless of its origin. However, many people have wondered why there isn’t a universal sign language for those who sign. This may be analogous to asking why there isn’t a universally accepted spoken language spoken all over the globe (Elakkiya, 2020).

Individuals who are deaf in Pakistan communicate with one another through the Pakistani Sign Language (PSL). It is subject to the rules of linguistics, just like all other sign languages, and, like the spoken Urdu language, it has its grammar, letters and words, and gestures and complex sentences. It also has a distinct vocabulary of signs and a constantly evolving syntax, just like any other sign language system worldwide. PSL has matured into a full-fledged language due to its evolution over time. Many people speak Urdu in South Asia, and it is the official language of Pakistan. Nastaleeq and Naskh are the most popular Urdu writing systems. It is extensively used in old Urdu literature and newspapers to write in the Nastaleeq way. Many other ethnic languages, including Persian, Pashto, Punjabi, Baluchi, and Saraiki, also use the Nastaleeq writing style to write their texts too. Indo-European language Urdu has its roots in India. It is one of the most widely spoken languages on the Indian subcontinent. Urdu is one of India’s 23 official languages and one of Pakistan’s two. Also, Dubai has a large population of people who speak this language. A majority of the world’s population speaks it. This is a written form of Urdu derived from the Persian script, which is evolved from the Arabic script. Urdu is also written from right to left, like Arabic. As a practical medium of interaction for deaf people everywhere, sign languages have emerged as the backbone of individual Deaf cultures. Hearing people who cannot communicate verbally due to a disability or disorder like augmentative and alternative communication or have deaf family members, such as children of deaf adults, utilize signs in addition to those who are deaf or have hearing loss. Furthermore, a blind person can also be benefitted from this work through a text-image to speech technology. If a character in an image is automatically detected through a machine, then converting it in the sound can be life-support to blind people.

**Figure 1:**
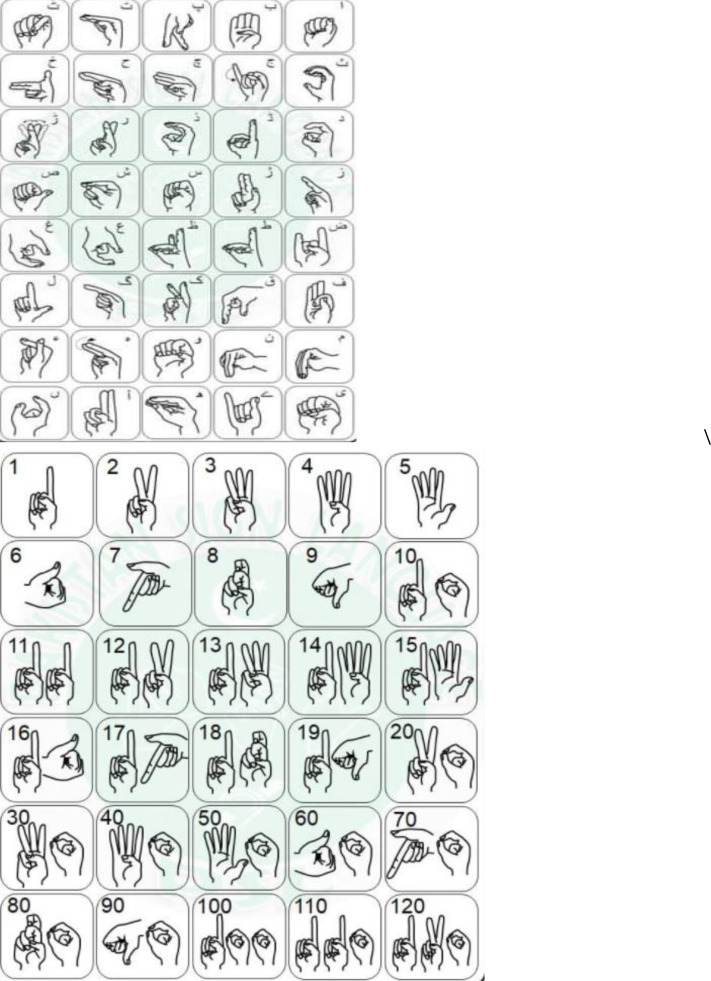
(A) Sign of Huroof-e- tahaji. (B) Sign of numbers (anonymous, b).

In contrast to Arabic and Persian, Urdu has more independent letters. Urdu has a more complex script than Arabic or Persian (Hussain, Ali & Akram, 2015; Anwar, Wang & Wang, 2006). In Figs. 1A and 1B, Urdu sign language is represented and labeled with words and numbers.

Gesture recognition has found a significant usage in this field, allowing deaf and mute patients to interact with us more efficiently and effectively. A considerable time and effort have been made in sign recognition worldwide. However, in the case of Urdu Sign Language, no such work could be found. Nearly 0.2 million deaf and mute Pakistani citizens do not have access to assistive and rehabilitative technology. There have been two types of gestures: static gestures are those gestures that incorporate dynamic hand, body, and face motions. Static gestures are those that do not change. During static gestures, the noticeable gesture occurs within a specific period that the performer physically orchestrates. A succession of finger and hand stances are identified and analyzed (Imtiaz et al., 2015; Subban & Mishra, 2013). In different parts of the world, other sign languages are used, including British Sign Language (BSL), American Sign Language (ASL), Arabic Sign Language (ArSL), and Spanish Sign Language (Carol & Humphries, 1988). Each of these sign languages has developed independently of the others. Typically, gestures in sign languages are generated either by signs that are ideographic notional hand movements, such as the thumbs-up, which is frequently used for the word “ok”, or by spelling words letter by letter following specific sign language norms (Li, Yang & Peng, 2009). Two key technologies are being deployed for hand posture or gesture recognition. There are two approaches: one is based on computer vision, which takes photographs of the signer and converts them into text using image analysis algorithms, and the other is based on machine learning. The third option is the use of a sensor-equipped glove (Oudah, Al-Naji & Chahl, 2020).

Due to various factors, the current state of Sign Language Recognition (SLR) is around 30 years behind voice recognition systems. One of the critical reasons for this is that receiving and detecting two-dimensional video data is far more complex than analyzing linear audio signals. Furthermore, verbal communication lexical and grammatical objects have yet to be fully discovered, and no conventional vocabularies are available. Aside from this, there are no traditional definitions for such a considerable number of signs. Sign language classification and recognition reached a high point in terms of research papers in the early 1990s (Elakkiya, 2020). The data collecting techniques are critical in categorizing the essential characteristics of various research on SLR. Due to the extreme dependability of sensor-based SLR systems, many studies have investigated data gloves or cyber gloves to extract the properties of the mechanical and non-mechanical components of the signs. Unfortunately, the usage of such sensors is unpleasant and restricting for the signer.

Furthermore, due to the high cost of sensors, real deployments of sensor-based SLR devices are impractical. On the other side, vision-based SLR systems have profoundly affected researchers due to their weight and capacity to handle crowded, dynamic heterogeneous situations and fluctuations under varied illuminations and occlusions in the feature extraction stage (Elakkiya & Selvamani, 2015; Elakkiya & Selvamani, 2018). The population sampling methods are critical in categorizing various SLR works’ essential aspects. Due to the extreme dependability of sensor-based SLR systems, many researchers have employed electronic gloves or cyber gloves to extract data of the mechanical and non-mechanical components of the signs.

Nonetheless, for the signer, the usage of these sensors is somewhat uncomfortable and extremely limiting (Elakkiya et al., 2012). In addition, due to the high cost of sensors, practical applications of sensor-based SLR systems are impractical. On the other hand, vision-based SLR systems have profoundly affected researchers due to their heaviness and capacity to manage crowded, dynamic heterogeneous surroundings and fluctuations in the segmentation stage under varying illuminations and occlusions (Elakkiya, Kannan & Selvamani, 2013). The SLR solutions’ standard element automatically allows signer-dependent actions, *i.e.,* all signers are trained before involving the patient. Signer independence or cross-validation among signers, on the other hand, entails the normalization of features to eliminate signer interactions. The range between some signers and the camera and the signer’s posture and magnification is rarely disclosed. SLR’s early phases were comparable to speech recognition in that they focused on individual signs.

Even though various SLR methods for identifying continuous phrases have been created, the detection accuracy has only achieved up to 90% for short dictionaries. The epenthesis motion occurs among adjacent signs in endless sign sentences. Previous studies have not specified if these are directly modeled, indirectly constructed, or just ignored. The action base will be expanded if transition movements are additionally simulated with the signs. Transition motions may be misclassified as signs unless they are modeled or ignored. The current recognition system recognizes a vast vocabulary simply utilizing sensor-based equipment, depending on the state-of-the-art sign language recognition. The classification performance is valid for the confined test situation, and many systems are signer-dependent. There isn’t much information about heftiness in real-time applications of SLR systems.

Furthermore, the specific vocabulary collections are unknown, and no common language for such speeches exists. In conclusion, none of the existing recognition systems meet the stringent real-world application requirements (Elakkiya & Selvamani, 2017). Keeping in mind the following implications of sensor-based SLR systems, the machine learning-based recognition seems too helpful and most effective and accurate.

On the other hand, computer vision-based methods use bare hands without colored gloves or sensors. Compared to sensor-based methods, vision-based solutions offer more mobility and normalcy for signers and be more cost-efficient due to a single camera. The classification methods may be divided into two types based on machine learning techniques: supervised learning and unsupervised learning. The SLR system can detect static and dynamic gestures of signs using these methods. For SLR, there are many categorization methods available. Neural networks (NNs), Hidden Markov models, support vector machine (SVM), KNN, K-means clustering, self-organizing maps (SOM), dynamic time warping, finite state machines, Kalman filtering, particle filtering, the condensation algorithm, and Bayesian classifier are some special classification techniques (Elakkiya, 2020; Gomes et al., 2016).

This literature review is needed to discover which classifiers have been utilized with what claimed accuracies and which sections of Urdu language have not been examined to locate data sources that other researchers have been using. To get insight into how others have defined and measured essential concepts. ‘Contribute to the advancement of knowledge in the area. It is a good idea to go over the literature to see what has been done previously and what worked and didn’t. Because of this, you may uncover gaps in the literature, which you can then seek to fix or address with your study by analyzing previous studies. To help future researchers in Urdu sign language, we have examined the merits and drawbacks of the previous research. This research study provides a literature review of all the previously published research studies in both journals (*n* = 10) and conference proceedings (*n* = 10), as shown in Tables 1 and 2, based on machine learning approaches for recognizing Urdu Sign language. This literature review will look at several published studies regarding their details, findings, and validity. We will explore them, summarize them, analyze them, and discuss them. Until July 2021, we will continue to conduct research based on research publications from databases such as PubMed, IEEE Xplore, ScienceDirect, and Google Scholar. The primary goal of this work is to evaluate the current efficacy of various machine learning approaches used to diagnose voice disorders and examine the development, weaknesses, and difficulties that have been identified and future research requirements. The following are the main contributions of this paper: (1) to review the classifiers, feature extracted, and accuracies of included articles. (2) To review the datasets and their types, no. of images, and accuracies of included articles. (3) Identify the gap.

**Table 1:** Detail conditions that are set for the adding and eliminating of published articles.

Conditions to add published articles	Conditions to eliminate published articles
Research articles that use Urdu sign language as a language for detection	Research articles that do not use Urdu sign language for detection.
Research articles that use machine learning classifiers as a problem solution.	Research articles that do not use machine learning classifiers as a problem solution
Research articles that report quantitative outcomes of machine learning.	Research articles that do not report quantitative outcomes of machine learning.
Research articles that only uses gesture-based, character bases, or EMG signal-based as a dataset to recognize Urdu Sign Language.	Research articles that do not use either gesture- based, character-based, or EMG signal-based as a dataset to recognize Urdu Sign Language.
Research articles that are written in the English language	Research articles that do not write in the English language.
Research articles that are published in Journals or conference proceedings.	Research articles that are published in either Journals or conference proceedings.

**Table 2:** Summarized detail of dataset for 20 included articles.

Author/Year	Dataset name	Dataset type	Dataset demographics	Publically available or not?	No. of images/ signals	Overall accuracy
Halim & Abbas (2014)	NA	PSL signs images	*n* = 10 (female = 4, male = 6)	No	NA	91%
Kanwal et al. (2014)	NA	PSL signs images	*n* = 30 (female = 15, male = 15)	No	NA	90%
Nasir et al. (2014)	NA	PSL signs images	NA	No	NA	97% ; 86%
Chandio et al. (2020)	NA	Text images from environment	NR	Yes	Isolated Urdu character image dataset contains 19901 images; cropped word image dataset contains 14100 words.	95%; 78.13%
Imran et al. (2021a)	NA	Signs of 37 characters of Urdu language	NA	Yes	40 images of each letter of Urdu language. 3˘7 x 40 = 1480 images.	90%
Naseem et al. (2019)	NA	Counting from 1 till 10	NA	No	Each sign’s dataset ranged from 1500 to 2000 images. The total number of imagesk was around 21000.	NA
Sagheer et al. (2010)	CENPARMI	Images of s isolated digits, numeral strings with/without decimal points, five special symbols, 44 isolated characters, 57 Urdu words (mostly financial related),	NR	No	14,407 samples images	97%
Ahmad et al. (2017)	UPTI	Images of sentences	NR	Yes	10063 sentence images	96%; 95%
Khan et al. (2020b)	NA	11 phrases of PSL using sEMG	NA	No	550 EMG signals	85.4%
Sami et al. (2014)	NA	37 Urdu alphabets	NA	No	NA	75%
Husnain et al. (2019)	NA	38 Urdu character and 10 numerals	500 native Urdu speakers	No	800 images (800 × 10 = 8000 numeral images and 800 × 38 = 30,400 Urdu characters)	96.04%; 98.3%
Khan et al. (2020a)	NA	26 alphabets in PSL using sEMG	NA	No	30 signals Per alphabet was recorded.	81%; 63%
Gul et al. (2020)	NA	NA	NA	No	NA	92.5%
Ahmad et al. (2007)	NA	NA	NA	No	NA	93.4%
Arafat & Iqbal (2020)	CLE	Synthetic image with ligature	NR	Yes	3801 training images and 423 test images.	94%
Ahmed et al. (2017)	UNHD	Sentences of Urdu language	500 writers	Yes	The dataset consist of 312,000 words written By 500 candidates with total of 10,000 lines.	92%

**Notes.**

PSL Pakistani Sign Language NA Not Available NR Not Required CENPARMI Centre for Pattern Recognition and Machine Intelligence UPTI Urdu Printed Text Images UNHD Urdu-Nasta’liq Handwritten Dataset

The following is a breakdown of the structure of this paper: “Introduction” includes a brief overview of sign language and discusses Urdu Sign Language in detail. The technique used to perform this literature review is described in “Materials and Methods”. The findings of this systematic examination are discussed in greater detail in “Results” of this document. “Discussion” discusses the primary research questions we are pursuing. “Conclusion” contains the conclusion of this entire work, including limitations, research gaps, and suggestions for further exploration.

## Materials and Methods

### Search methodology

For this literature review, the population (P), intervention (I), comparison (C), and outcome (O) base PICO method was taken into consideration which was previously used by Syed, Rashid & Hussain (2020) and clearly defined the goals and intervention of this literature review and for what population it is intended for. PICO was used to develop the search strategy, which was as follows: Population = deaf people in Pakistan, Intervention = recognition of Urdu Sign Language, Comparison = all the datasets developed for Urdu Sign Language and all the machine learning classifiers implemented on Urdu Sign Language, and Outcome = accuracies reported in the selected study. To construct a set of search strings, the Boolean operator combined relevant analogs and alternative words: AND focuses and limits the search, while OR widens and increases the investigation (Syed, Rashid & Hussain, 2020). The following search term was created with the assistance of these Boolean operators:

• (Pakistani sign language) OR (Urdu sign language) AND (“computer vision” OR “neural network” OR “artificial intelligence” OR “pattern recognition” OR “machine learning”)
• (Pakistani sign language) AND (“computer vision” OR “neural network” OR “artificial intelligence” OR “pattern recognition” OR “machine learning”)
• (Urdu sign language) AND (“computer vision” OR “neural network” OR “artificial intelligence” OR “pattern recognition” OR “machine learning”)
• (Urdu sign language/Pakistani sign language) AND (computer vision)
• (Urdu sign language/Pakistani sign language) AND (neural network)
• (Urdu sign language/Pakistani sign language) AND (artificial intelligence)
• (Urdu sign language/Pakistani sign language) AND (pattern recognition),
• (Urdu sign language/Pakistani sign language) AND (machine learning)

**Figure 2:**
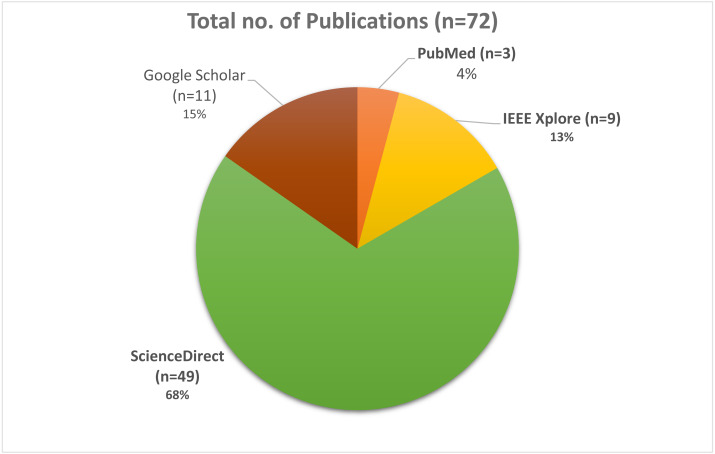
Pie Chart depicts the total number of research studies.

**Figure 3:**
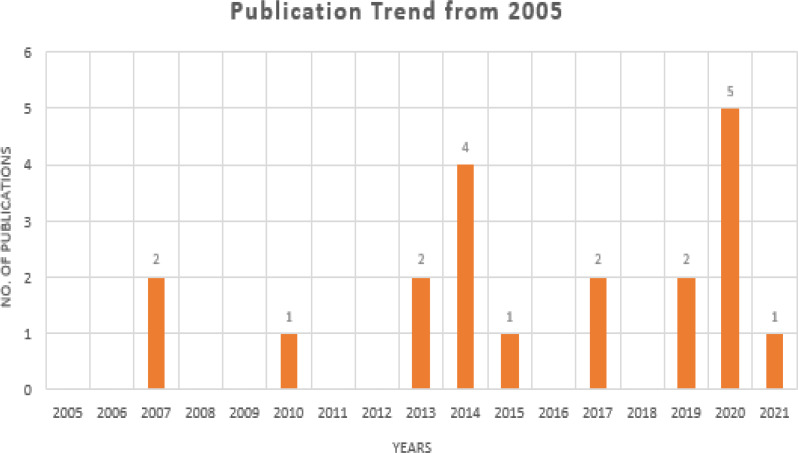
Publication trends in the last 17 years.

Searches for peer-reviewed papers were conducted in four large databases: PubMed, IEEE Xplore, Google Scholar, and ScienceDirect (all of which are free to use). Review papers, research articles, conference abstracts, correspondences, data articles, debates, and case reports were the only types of articles that could be found in ScienceDirect. All three databases will be checked until July 2021. These databases have been searched using a set of keywords that have been shown above and utilized to do searches. The search results were found in PubMed (*n* = 3), IEEE Xplore (*n* = 9), Google Scholar (*n* = 11), and ScienceDirect (*n* = 49), with a total of 72 results when the initial search was performed, as shown in Fig. 2. In Fig. 3, it can be seen that most research work for the application of machine learning techniques for Urdu Sign Language Recognition has been conducted and published in the last fifteen years, from 2007 to 2021, which itself exhibits the significance of this investigation.

### Survey methodology

**Figure 4:**
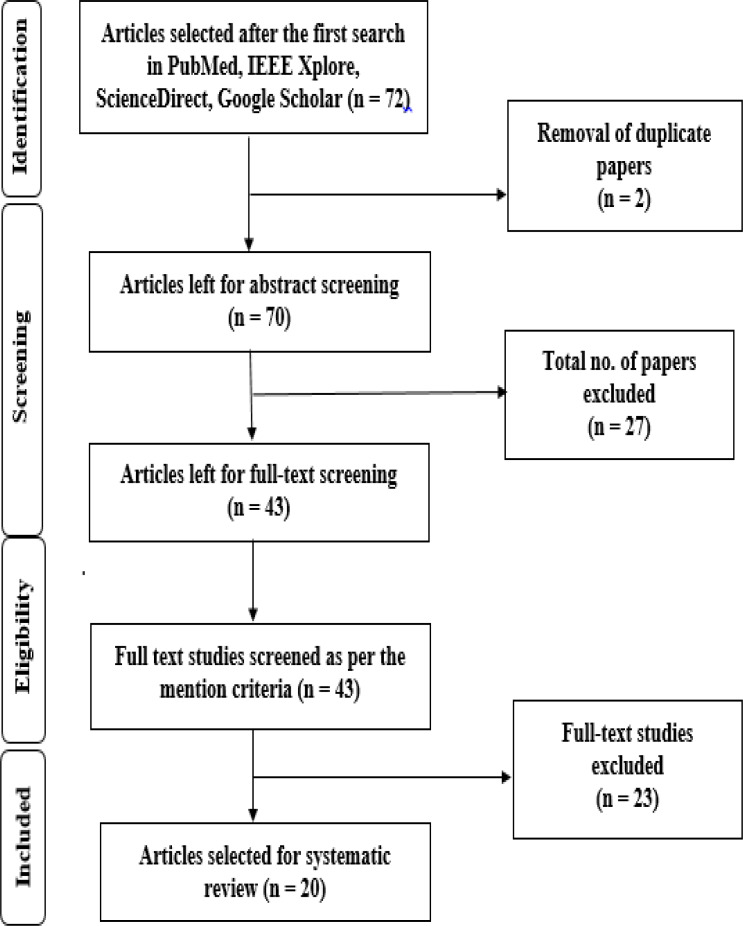
Flowchart as per PRISMA (Liberati et al., 2009) guidelines.

To declutter the research studies extracted through the PICO search method defined in Syed, Rashid & Hussain (2020), PRISMA (Liberati et al., 2009) protocols were followed. Search results were collected and arranged using the online endnote system, as depicted in Fig. 4. The endnote web system constructed a data table taken from each selected document. Full texts of articles that were deemed possibly appropriate were uploaded to the Endnote website for viewing (by Clarivate Analytics). In the first attempt, it was necessary to apply the search criteria (Table 1) to each specified database to include the whole document in journals and conferences. There have been thousands of useless results from this approach, and as a result, a decision is taken to limit further the search to only the title and type of material contained within the page.

Further research is determined by referring to the sources of the linked studies discovered. Following the collection of leading search research, we evaluated the titles and abstracts of the studies to identify relevant ones. The current investigation results, including a comprehensive text, are being used to assess the relevant studies.

## Results

**Figure 5:**
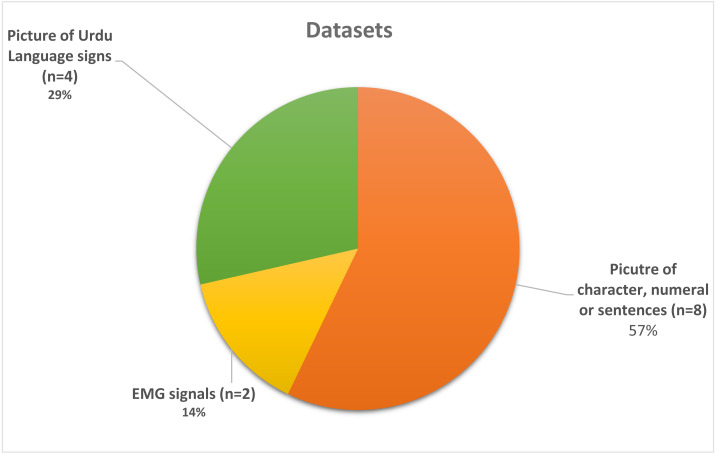
Pie chart mainly representing the used dataset.

**Figure 6:**
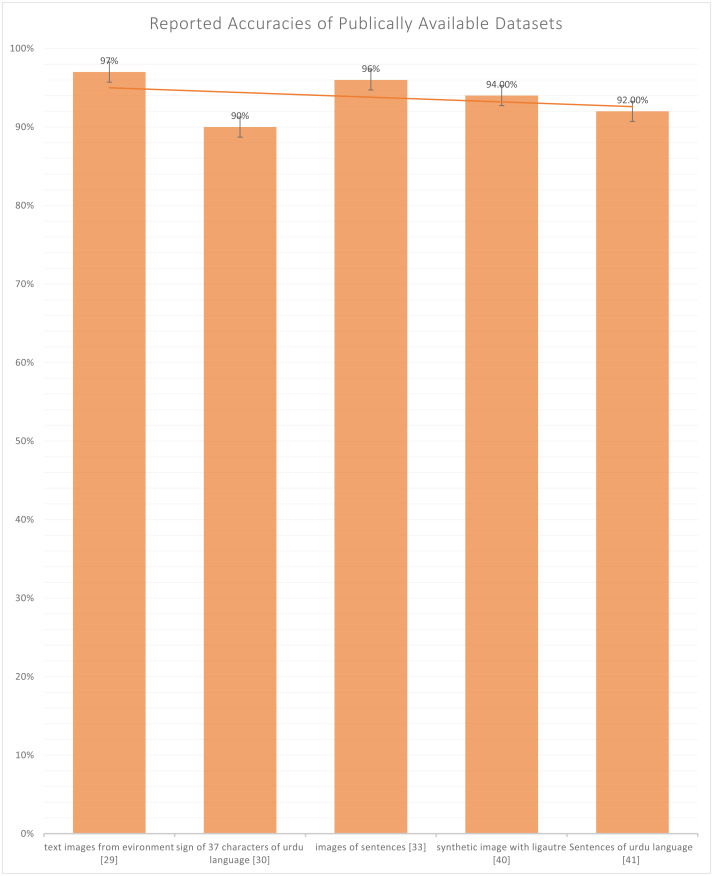
Graph of reported accuracies of the publicly available dataset.

Table 2 depicts the details of all the datasets used in the selected studies, including a character-based, EMG signals-based, and sign images-based dataset. Figure 5 representing that which dataset is the most used and which is the least used and as per the resulting signal based EMG data has only used twice in Khan et al. (2020b) and Khan et al. (2020a) whereas dataset which contains the images of sign used four times in Halim & Abbas (2014), Kanwal et al. (2014), Nasir et al. (2014) and Imran et al. (2021a). The most used type of dataset are the character-based datasets in Chandio et al. (2020), Naseem et al. (2019), Sagheer et al. (2010), Ahmad et al. (2017), Sami, (2014), Husnain et al. (2019), Gul et al. (2020) Arafat & Iqbal (2020) and Ahmed et al. (2017). Also, in Fig. 6, we can observe that only five datasets are publically available out of four datasets (Chandio et al., 2020; Sagheer et al., 2010; Arafat & Iqbal, 2020; Ahmed et al., 2017) contain either character, numeral, or sentence-based images. Only one dataset (Liberati et al., 2009) is publically available, which is based on images of the visually impaired individual making signs of Urdu Language and the highest accuracy reported in publically available datasets is 97% by Chandio et al. (2020), which is reported on the text-based dataset and not on sign based dataset which is the actual lackness in this area.

**Figure 7:**
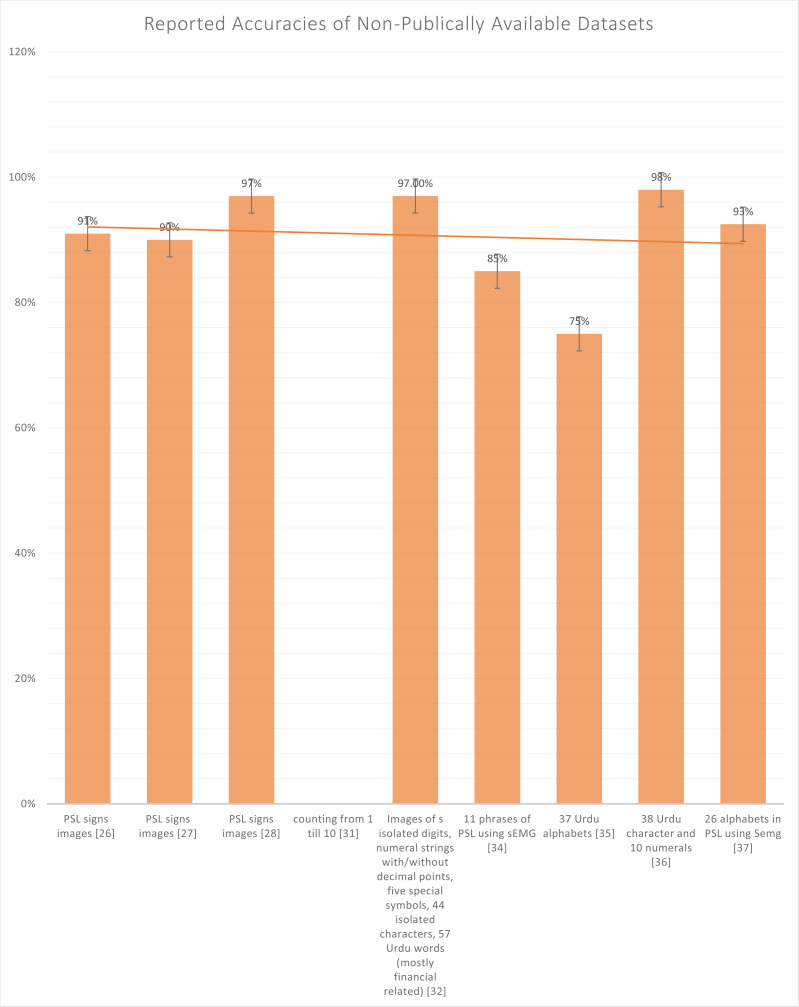
Graph of reported accuracies of the dataset that is not publicly available.

Whereas in Fig. 7, all the not publically available datasets, the highest reported accuracy 98%, again reported on the text-based dataset and not on the sign-based dataset. In Table 2, Halim & Abbas (2014), Kanwal et al. (2014), Nasir et al. (2014) and Imran et al. (2021a) are the only authors who have used sign based language dataset. Generally, there are two types in recognizing the Urdu language, *i.e.,* sign-based images and text-based images. Sign language recognition models employ two kinds of input data to extract the essential characteristics: static and dynamic. Several deep-based models using still or sequential inputs have been presented in recent years. While active inputs provide sequential information that might help increase the sign language recognition rate, there are still certain obstacles to overcome, such as the computational cost of input sequences. Dynamic inputs may also be divided into separate dynamical inputs and continuous inputs. Discrete active inputs are utilized at the word level, whereas continual inputs are used at the sentence level. Tokenization of sentences into individual words, identifying the start and conclusion of a phrase, and handling abbreviations and repetitions in the sentence are all issues with continuous dynamic inputs. We’ll go through the sign language recognition algorithms that have utilized these inputs in the following sections.

Table 3 summarizes all 20 studies that are included in this literature review. This table covers all the important details, *i.e.,* classifier name, which feature is extracted, reported accuracy, reported sensitivity and reported specificity. The study is either published in a journal or conference. From Table 3, we can analyzed that the SVM (Chandio et al., 2020; Imran et al., 2021a; Sagheer et al., 2010; Ahmad et al., 2017; Khan et al., 2020b; Ahmed et al., 2017; Imran et al., 2021b) and Neural Network (Chandio et al., 2020; Naseem et al., 2019; Ahmad et al., 2007; Arafat & Iqbal, 2020; Sagheer et al., 2009; Naz et al., 2015; Ul-Hasan et al., 2013) is the commonly used classifier by researchers for the detection of Urdu Sign Language other than these both rest of the classifiers used only once *i.e.,* DTW (Halim & Abbas, 2014), HMM (Gul et al., 2020).

**Table 3:** Summarized detail of machine learning classifiers and feature extraction of 20 included articles.

Author/Year	Journal/ Conference	Dataset	Classification technique	Feature selection/filter	Overall accuracy	Overall sensitivity	Overall specificity
Halim & Abbas (2014)	Journal	NA	DTW	Normalization of skeleton frame data	91%	NA	NA
Kanwal et al. (2014)	Conference	NA	Euclidean Distance	PCA coefficients	90%	NA	NA
Nasir et al. (2014)	Conference	NA	Bagging Ensemble	Dimension reduction by PCA	97%; 86%	NA	NA
Chandio et al. (2020)	Journal	NA	SVM; RNN-CNN	HOG, LBP; CTC	95%; 78.13%	NA	NA
Imran et al. (2021a)	Journal	NA	SVM	Saturation and Hue components of image	90%	NA	NA
Naseem et al. (2019)	Journal	NA	CNN	Inception V3 architecture	NA	NA	NA
Sagheer et al. (2010)	Conference	CENPARMI	SVM	Structural and gradient features	97%	NA	NA
Ahmad et al. (2017)	Journal	UPTI	ULR-SDA; SVM	jitter, elastic elongation, threshold and sensitivity	96%; 95%	NA	NA
Khan et al. (2020b)	Conference	NA	Linear SVM	Time domain, spectral domain, shape, and texture	85.4%	85.36%	85.81%
Sami et al. (2014)	Conference	NA	Cross correlation	Edge detection of second derivative	75%	NA	NA
Husnain et al. (2019)	Journal	NA	CNN	Pixel- and geometrical-based	98.3%	NA	NA
Khan et al. (2020a)	Conference	NA	Linear Discriminant; Quadratic discriminant	Time domain, statistical domain, shape, spectral domain, cepstral domain	81%; 63%	84.05%; 66.4%	84.7%; 65.9%
Gul et al. (2020)	Conference	NA	HMM	3D vectors	92.5%	NA	NA
Ahmad et al. (2007)	Journal	NA	NN	Segmentation of characters	93.4%	NA	NA
Arafat & Iqbal (2020)	Journal	CLE	DLN (FasterRCNN, RRNN, TSDNN)	Resnet50, Googlenet	94%	NA	NA
Ahmed et al. (2017)	Conference	CENPARMI	SVM	400D gradient feature	98.61%	NA	NA
Sagheer et al. (2009)	Journal	UNHD	RNN	Textline segmentation	92%	NA	NA
Sabbour & Shafait (2013)	Conference	UPTI	Line segmentation	Contour extraction, shape context	91%	NA	NA
Naz et al. (2015)	Journal	UPTI	MD-RNN	vertical and horizontal edges intensities, foreground distribution, density function, intensity feature, horizontal projection, contrast intensity	94.97%	NA	NA
Ul-Hasan et al. (2013)	Conference	UPTI	BLSTM-RNN	Baseline information	86.42%	NA	NA
Imran et al. (2021b)	journal	NA	SVM	Achromatic decomposition	90%	NA	NA

**Notes.**

PSL Pakistani Sign Language NA Not Available NR Not Required DTW Dynamic Time Warping PCA Principal Component Analysis SVM Support Vector Machine HOG Histogram of Oriented Gradients LBP Local Binary Pattern CTC Connectionist Temporal Classification RNN Recurrent Neural Network CNN Convolutional Neural Network CENPARMI Centre for Pattern Recognition and Machine Intelligence UPTI Urdu Printed Text Images ULR-SDA Urdu Ligature Recognition Stacked Denoising Auto encoder EMG Electro MyoGraphy HMM Hidden Markov Model NN Neural Network DLN Deep Learning Network RRNN Regression Residual Neural Network TSDNN Two Stream Deep Neural Network CLE Center of Language Engineering UNHD Urdu-Nasta’liq Handwritten Dataset MD-RNN Multi-Dimensional Recurrent Neural Network BLSTM Bidirectional Long Short Term Memory

**Figure 8:**
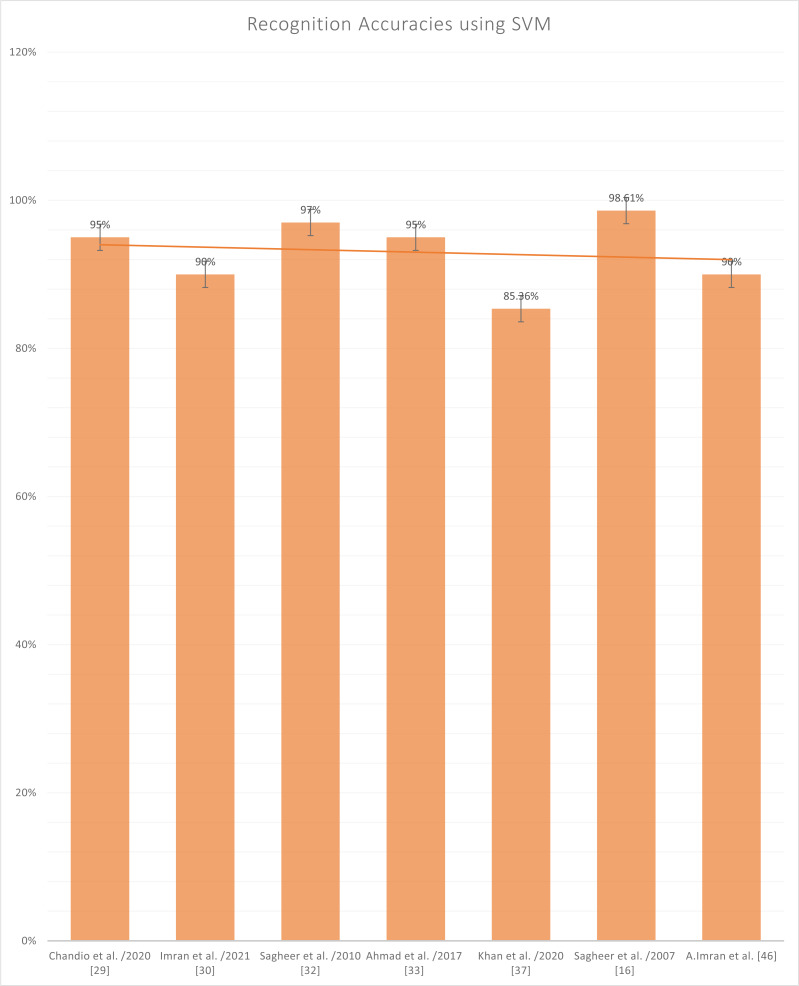
Graph of reported accuracies of detection of Urdu Sign Language using SVM.

Figure 8 represents the reported accuracies that have been generated after using SVM as a classifier, and it has been noted that the highest accuracy in SVM is written by Sagheer et al. in (Ahmed et al., 2017) on a character-based dataset which is publically available by the name of UNHD (Urdu-Nasta’liq Handwritten Dataset). The Support Vector Machine (SVM) is an old classification technique that has piqued the research community’s attention, particularly in machine classification, regression, and learning, among other areas. SVM with the accompanying classes that are well-known. This is described as the process of filtering or extracting characteristics. Even if no prediction of unknown samples is required, feature evaluation and SVM classification have been utilized in conjunction with one another. In class differentiation, they can designate the main sets involved in the process. The SVM depicts the entrance space as a vast area with several doors. By generating an optimal hyperplane separation, the SVM determined the boundary between regions belonging to both classes of sites. The hyperplane is selected to optimize the separation between the closest samples of exercises. Initially, SVM models were developed to sort linear categories into subcategories. Because of the massive characteristics, it is impossible to use the function attributes to identify the separation hyperplane in their pure form.

The specific part is used to calculate non-linear mapping utilizing unique non-linear variables called the kernel, derived from the characteristic function. It has the advantage of operating in the input area where the weighted sum of the kernel function assessed by support vectors can be utilized to solve the classification problem. In contrast, the support vectors have the disadvantage of only functioning in the output area. The SVM algorithm can create a variety of learning machines by utilizing a variety of kernel functions. Compared to artificial neural networks, SVM tends to be significantly more accurate and produce more promising outcomes (Uma Rani & Holi, 2014). Support vector machines (SVMs) have emerged as a popular machine learning technique for classification, regression, and novelty detection tasks. They exhibit outstanding performance and effectiveness on a wide range of real-world questions, and the approach is conceptually motivated by logic. It is not necessary to seek out the architecture of the learner machine through experimentation (Huang et al., 2018) to achieve success. There are only a few free parameters available. Even though SVMs are incredibly effective classifiers that use non-linear kernels, they have drawbacks: (1). It is necessary to test alternative kernel configurations and model parameters to obtain the optimal model; (2).

In some cases, training might take a lengthy time, especially if there are many characteristics or examples in the data set; (3). Their inner workings are difficult to comprehend since the fundamental models are built on sophisticated mathematical frameworks, and their conclusions are tough to interpret. The selection of features using all available data, followed by the testing of classifier training, for example, results in an optimistic error estimate (Yue, Li & Hao, 2003).

**Figure 9:**
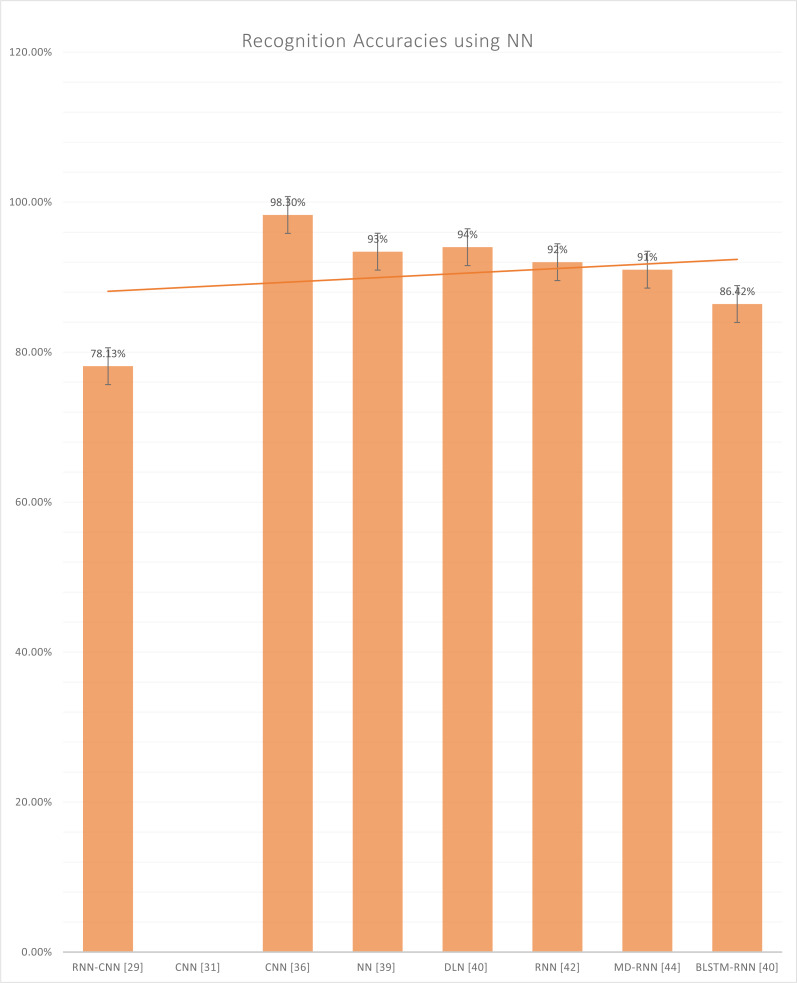
Graph of reported accuracies of detection of Urdu Sign Language using Neural Network.

Figure 9 represents the reported accuracies that have been generated after using neural networks, which include RNN, CNN, DLN, MD-RNN, and BLSTM-RNN. In the CNN, numerous hierarchy levels are formed of routing groups and grouping layers, and each of these levels is defined by a different type of chart. A convolutional layer, which receives data at the input level, is the starting point for most CNNs. The convolution layer is responsible for convolutionary processes involving a small number of filtering maps of the same dimension. In addition, the result from this layer is passed to the sample layer, which reduces the scale of the subsequent layers in the sequence. CNN is closely associated with a wide range of deep neural networks locally (Jan et al., 0000). These systems are then deployed on several hundred cores of GPU architecture based on the GPU architecture. Following the previous layer information blocks (O’Shea & Nash, 2015), the appropriate people will assign the role maps. It is dependent on the size of the maps.

On the other hand, each thread is tied to a single neuron utilizing a suitable block that contains several lines. Similarly, neuron convolution, induction, and summation are performed on the input neurons throughout the procedure. Finally, the techniques described above are stored in global memory. A reverse and propagation model is used to handle results as efficiently as possible. On the other hand, pulling or moving activities lead to parallel spread because a single distribution would not result in a beneficial consequence. As mentioned previously, the neurons of a single layer communicate with a different number of neurons, which impacts the border effect (Yang & Horie, 2015).

**Figure 10:**
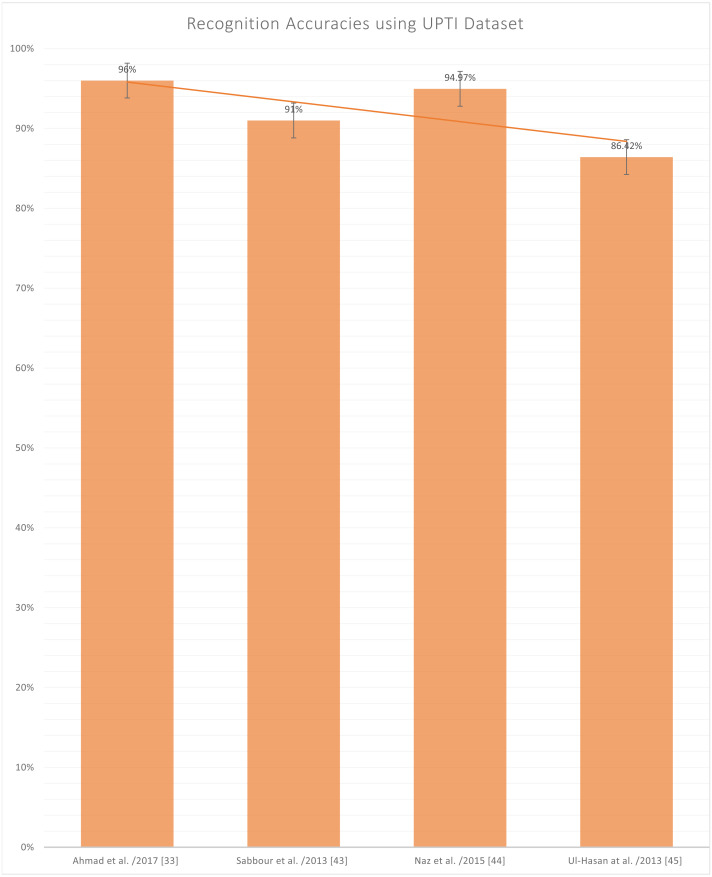
Graph of reported accuracies for detection of Urdu Printed Text Images.

Figure 10 represents the reported accuracies using the UPTI (Urdu Printed Text Images) dataset (Ahmad et al., 2017; Sabbour & Shafait, 2013; Naz et al., 2015; Ul-Hasan et al., 2013), as it can be observed in Table 2 that UPTI is the most used dataset and the highest reported accuracy is 96%. This dataset contains a total of 10,063 images of different sentences of the Urdu language, and this is also a publically available dataset. But the problem lies here that this is not the sign language dataset, which is the authors’ primary concern.

## Discussion

The author’s first concern is the lack of publicly available datasets containing images of individuals making the signs. Only one such dataset is available in Mendeley by Imran et al. in Sami (2014) in 2021, which is very recent. There are so many things that can be pointed out as a flaw. The number of participants used for this dataset is 40. Each individual contributes 37 pictures (one picture each of 37 Urdu characters), making only 1480 images in total considered a minimal dataset. Furthermore, only SVM as a classifier to validate this dataset.

Another lack of concern that the author has is the lackness of machine learning outcomes, *i.e.,* specificity and sensitivity. Only one study, *i.e.,* Khan et al. (2020a), reports all three products: accuracy, specificity, and sensitivity. Naseem et al. (2019) didn’t write the accuracy, the primary machine learning outcome. The last noticeable thing is that not even a single author has used unsupervised techniques as a classifier in the screened studies, which means that a lot of work needs to be done in this area.

When it comes to the limitations of this literature review, we can’t ignore the fact that the number of papers included was far lower than expected. As a second point, only studies published in English were considered for inclusion, limiting the representation of work from non-English countries that speak and the generalizability of the findings. Third, there is a strong likelihood that the search technique used for this review overlooked some significant articles, given that papers published in conference proceedings were primarily disregarded.

## Conclusion

When a person cannot hear, they are deaf, making communicating with others extremely difficult. More than 5% of the global population, including adults and children, is deaf, and around 10 million Pakistanis are deaf. Another impairment is muteness, which occurs when an individual cannot talk or communicate correctly. There are many other types of disabilities. People like this have a very distinct manner of connecting with the rest of the world. Through “Sign Language”, they communicate their feelings and thoughts to the rest of the world. Sign language is very distinct and not comprehended by others; many institutions and organizations worldwide teach people with disabilities and their families sign dialects to make one’s lives more accessible; however, learning sign language is not easy. Not everybody is familiar with it. This literature review summarized 20 screened studies included after the detailed screening. It is also concluded that SVM and Neural Network are the most common classifiers. The first identified gap is the lack of publically available datasets and, most specifically, datasets with images of signs of Urdu characters and not the actual characters. The second identified gap is that the authors can use unsupervised machine learning classifiers because this is an untouched territory, and a tremendous amount of work can be done here.

## Additional Information and Declarations

### Data Availability

The following information was supplied regarding data availability: This is a literature review.
